# Patient‐reported physical well‐being predicts good long‐term survival of hematopoietic stem cell transplantation

**DOI:** 10.1002/cam4.7409

**Published:** 2024-07-05

**Authors:** Yin Lu, Tao You, Qin Ma, Weijuan Wang, Jiaqian Qi, Pan Yang, Yaya Song, Jia Chen, Jianhong Fu, Yongqin Ge, Xiaming Zhu, Depei Wu

**Affiliations:** ^1^ National Clinical Research Center for Hematologic Diseases Suzhou China; ^2^ Department of Hematology The First Affiliated Hospital of Soochow University Suzhou China; ^3^ Hematopoietic Transplant Institute, Soochow University Suzhou China; ^4^ Jiangsu Institute of Hematology, The First Affiliated Hospital of Soochow University Suzhou China; ^5^ Collaborative Innovation Center of Hematology Soochow University Suzhou China; ^6^ Shandong Province Qianfoshan Hospital Jinan Shandong China

**Keywords:** hematological malignancy, hematopoietic stem cell transplantation, predict, quality of life, survival

## Abstract

**Aim:**

This study aimed to explore the association between patient‐reported items at different time points after hematopoietic stem cell transplantation (HSCT) and long‐term survival.

**Methods:**

We conducted a study with 144 allogeneic HSCT patients, following them for 5 years post‐transplantation. Data from the Functional Assessment of Cancer Therapy‐Bone Marrow Transplant (FACT‐BMT) questionnaire were collected before transplantation and at 1, 3, 6, 12, 18, 36, and 60 months after transplantation. Demographic characteristics and survival status were also assessed.

**Results:**

Among the 144 cases, the 5‐year overall survival (OS), progression‐free survival (PFS), non‐relapse mortality (NRM), and graft‐versus‐host disease‐free (GRFS) rates were 65%, 48%, 17%, and 36% respectively. Health‐related quality of life (HRQOL) showed a fluctuating pattern over 5 years. Using a latent class mixed model, patients were classified into two groups based on their physical well‐being (PWB) scores during the 60‐month follow‐up. Class 1 had initially lower PWB scores, which gradually increased over time. In contrast, Class 2 maintained higher PWB scores with slight increases over time. Kaplan–Meier survival analysis revealed that Class 1 had better OS (70.9% vs. 52.9%, *p* = 0.021), PFS (60.5% vs. 41.2%, *p* = 0.039), and GRFS (35.1% vs. 29.3%, *p* = 0.035) compared to Class 2.

**Conclusions:**

Patients who had higher initial PWB scores after HSCT demonstrated improved long‐term survival outcomes. The PWB score could serve as a valuable predictor for the prognosis of HSCT.

## 1 INTRODUCTION

Allogeneic hematopoietic stem cell transplantation (Allo‐HSCT) is a powerful therapeutic strategy for patients diagnosed with high‐risk hematologic malignancies.^1^, ^2^ Annually, over 60,000 individuals worldwide undergo either allogeneic or autologous HSCT, leading to an increase in survivorship.^3^ However, long‐term morbidity and late mortality continue to present significant challenges.^4^ Therefore, the ongoing improvement and enhancement of patients' quality of life (QOL) following HSCT remain a central focus.

In recent years, researchers have developed various prognostic tools aimed at improving the prediction of post‐transplant outcomes.^5^, ^6^, ^7^, ^8^ For example, the Hematopoietic Cell Transplantation‐Composite Risk (HCT‐CR), which incorporates the refined disease risk index (DRI‐R) and the hematopoietic stem‐cell transplant comorbidity/age index (HCT‐CI/Age), has been shown to more accurately predict the risk of death associated with Allo‐HSCT compared to conventional models for patients diagnosed with acute myeloid leukemia (AML) and myelodysplastic syndrome (MDS).^2^

Despite patient‐reported outcomes (PROs) being recognized as predictors of mortality in various cancer types, their consideration within the HSCT field remains limited.^9^ In HSCT, over 90% of survivors experience at least one severe late adverse effect resulting from the treatment.^10^ Consequently, thorough assessment and documentation of patients' QOL have become increasingly important in clinical management. Furthermore, QOL has been acknowledged as a predictor of response to future treatments and is widely utilized in clinical trials.^11^, ^12^, ^13^, ^14^

QOL is a comprehensive concept that can change over time based on individual assessments and environmental factors, encompassing various aspects of patients' experiences.^15^, ^16^ Allo‐HSCT patients typically undergo distinct treatment stages, during which their QOL may vary.^17^ Although our previous research identified the trajectory of QOL in the early stages of HSCT, the long‐term progression of QOL among survivors and whether early‐stage QOL can effectively predict survival outcomes remain unclear. Therefore, we conducted a 5‐year follow‐up study with Allo‐HSCT patients, examining their QOL at 12, 18, 36, and 60 months post‐transplantation to determine their long‐term QOL trajectories and explore the relationship between HSCT treatment efficacy and QOL.

## 2 METHODS

### 2.1 Participants

We conducted a retrospective analysis of patients who underwent HSCT between July 22, 2016, and April 30, 2017, at the First Affiliated Hospital of Soochow University in China. Patients were included if they were 18 years old or older and were willing to participate in the study. Patients with mental illness or cognitive impairment were excluded, as were those illness was recrudescent, who were too sick to participate, or who die. These patients had previously participated in our investigation and completed assessments before transplantation, as well as at 1, 3, and 6 months post‐transplantation, totaling 191 patients were enrolled. (Results can be found in Liang YC.^3^) Patients who could not be stratified according to the disease risk index model criteria and those who underwent autologous HSCT were excluded. We also excluded five patients for insufficient following up information. Finally, we followed up with 144 patients and assessed their QOL at 12, 18, 36, and 60 months post‐transplantation, respectively. This study was approved by our hospital's ethics committee (IRB no. 2017021), and all subjects provided written informed consent in accordance with the Declaration of Helsinki.

### 2.2 The Functional Assessment of Cancer Therapy—Bone Marrow Transplant

We assessed QOL using the Functional Assessment of Cancer Therapy—Bone Marrow Transplantation (FACT‐BMT), Simplified Chinese Version 4.0.^17^, ^18^ This assessment includes five domains: physical well‐being (PWB), social/family well‐being (SWB), emotional well‐being (EWB), functional well‐being (FWB), and BMT‐specific concerns (BMTS). Participants rated their responses on a scale from 0 to 4 (0 = not at all; 1 = a little bit; 2 = somewhat; 3 = quite a bit; 4 = very much). The scores from these five domains were combined to generate a total score, with reliability coefficients ranging from 0.86 to 0.89.^18^ Yan X and colleagues confirmed that FACT‐BMT is a reliable measure of QOL for Chinese HSCT patients.^19^ Higher scores indicate better QOL.

### 2.3 Clinical data collection

Data concerning baseline clinicopathological features, disease information, pre‐HSCT disease status, HSCT type, conditioning regimens (busulfan and cyclophosphamide or others), platelet levels (measured upon patients' exit from the protective environment), neutrophil engraftment time, and overall survival (OS) outcomes were collected from each patient's medical records. This information was obtained from the HSCT follow‐up team responsible for monitoring HSCT patients within the department of hematology.

### 2.4 Statistical analysis

Numerical data with normal or skewed distributions were presented as $\bar{x}$ ± SD or median with interquartile ranges and compared using Student's *t*‐test or the Mann–Whitney *U*‐test. Categorical data were presented as numbers with percentages and compared using the chi‐square test or Fisher exact method, as appropriate. A latent class mixed model was employed to divide patient groups based on PWB score dynamics. The Kaplan–Meier curve with the log‐rank test was utilized for survival analysis. A multivariate Cox model was applied to estimate survival probabilities according to PWB class. Multiple imputation was used for missing value analysis.

For latent profile analysis, two different latent class growth models of PWB were estimated: the first fitting only one latent profile, the second two profile groups. Each model included terms for linear time effects according to the 8 time point design. The estimated latent profile group with the higher BIC was selected.^20^ Statistical analyzes were performed using the survival, survminer, and LCMM packages in R software (Version 4.0.3, Vienna, Austria).

## 3 RESULTS

### 3.1 Patients' characteristics

A total of 144 patients were analyzed, 69 patients diagnosed AML, 42 patients were acute lymphocytic leukemia, 25 patients were MDS, 3 patients were lymphoma and 3 patients were chronic myeloid leukemia. Among all the patients, the 5‐year OS was 65%, and the progression‐free survival (PFS), non‐relapse mortality (NRM), and graft‐versus‐host disease‐free (GRFS) rates were 48%, 17%, and 36%, respectively. Analysis all the survival patients after 5 years transplantation, there were 59.8% patients had already back to normal living status. The association of baseline clinical features with OS outcome was displayed in Table S1.

### 3.2 Relationship between FACT‐BMT and survival outcomes

No clinical covariates were found to be predictive of survival.

### 3.3 Definition of two patient classes based on PWB score

**FIGURE 1:**
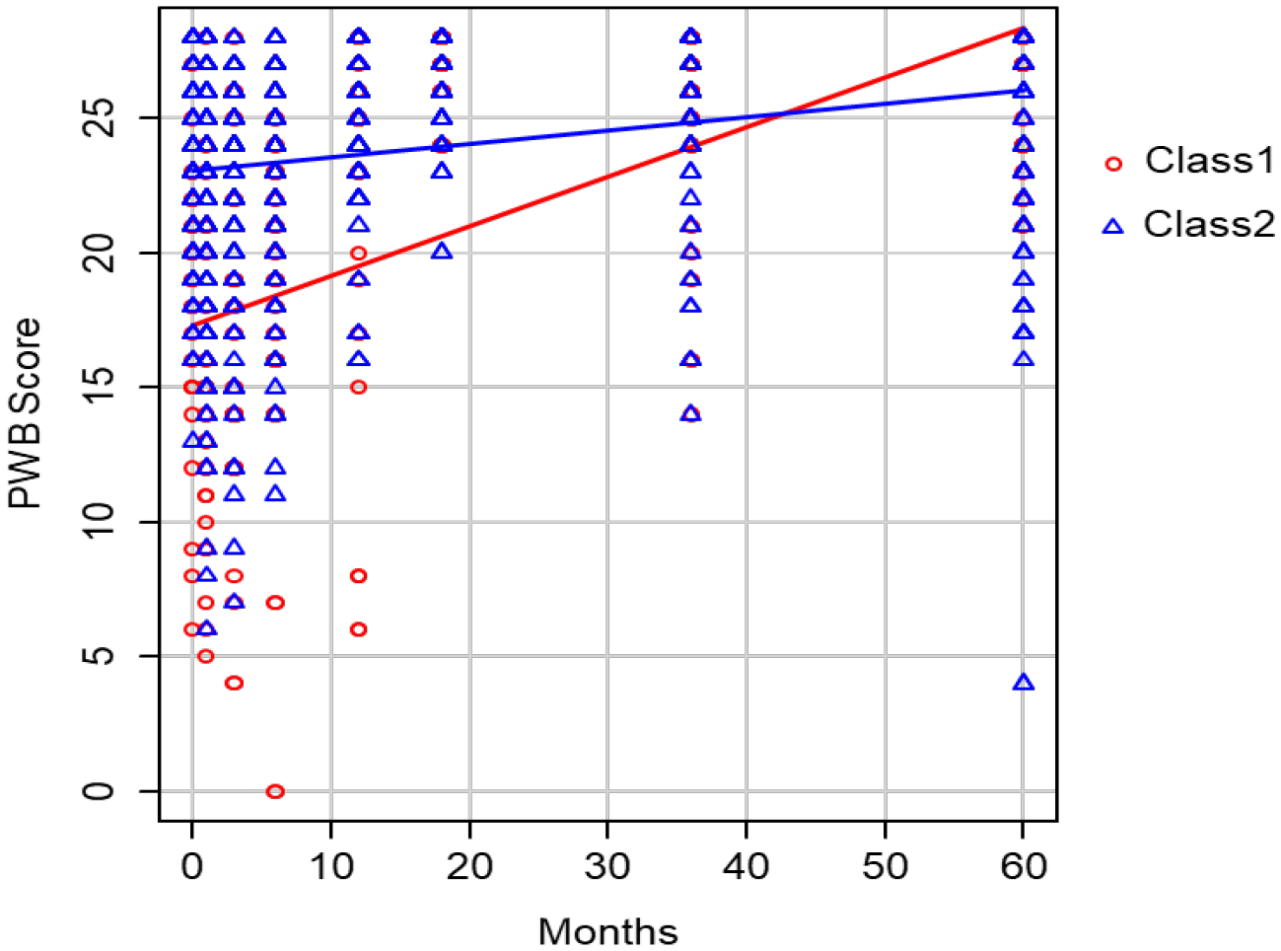
Two group latent class analysis of PWB trajectories through 60 months follow up.

A latent class mixed model was utilized to divide HSCT patients into two groups based on PWB scores throughout the 60‐month follow‐up period. Results revealed that the first class (Class 1) exhibited a lower PWB score during the initial 48 months post‐HSCT, with a gradual increasing trend observed. In contrast, the second class (Class 2) maintained a slower PWB level increase throughout the follow‐up period and showed a lower PWB compared to Class 2 after 48 months (Figure 1). Their baseline characteristics and information of transplantation are presented in Table 1. Most clinical characteristics were similar between PWB Class 1 and Class 2 patients.

**TABLE 1:** Baseline demographic and medical data of the participants in the study.

	Total *N* = 144	Class 1 *n* = 27	Class 2 *n* = 117	*X* ^2^	*p*‐value
Age	32.91 ± 11.7	33.04 ± 9.17	32.88 ± 12.25	6.322	0.941
Sex					
Female	50	12	38	1.386	0.239
Male	94	15	79		
Marriage					
Spinsterhood	48	8	40	0.44	0.775
Married	94	19	75		
Divorce	2	0	2		
Residential address					
City	45	11	34	1.646	0.674
Town	25	3	22		
County	23	4	19		
Village	51	9	42		
Education					
Master	2	1	1	2.47	0.633
Bachelor	32	7	25		
High school	67	13	54		
Junior school	37	6	31		
Primary school	4	0	4		
House incoming					
More than 5001	36	5	31	4.687	0.31
4001–5000	17	1	16		
3001–4000	25	4	21		
2001–3000	27	6	21		
Lower than 2000	39	11	28		
Source of hospitalization					
Medical insurance	125	26	99	2.613	0.126
Self‐paying	19	1	18		
Previous history					
None	118	21	97	1.894	0.721
Hypertension	4	1	3		
Diabetes	3	1	2		
Hepatitis B	6	1	5		
Others	13	3	10		
Diagnosis					
AML	69	14	55	1.424	0.861
ALL	42	7	35		
MDS	25	4	21		
Lymphoma	3	1	2		
CML	5	1	4		
Disease risk index					
Low	9	2	7	2.514	0.259
Intermediate	67	9	58		
High	68	16	52		
Type of HSCT					
Matched‐related	46	14	32	5.675	0.05
Haplo‐	84	11	73		
Unrelated‐	14	2	12		
Induced program					
BUCY	115	21	94	1.205	0.572
Decitabine + BUCY	23	4	19		
Others	6	2	4		
Duration of living after HSCT	44.8 ± 23.33	35.59 ± 27.48	46.92 ± 21.86	12.673	0.053
Complication in HSCT (including GVHD)					
Yes	137	26	111	0.096	0.756
No	7	1	6		
Complication before HSCT					
Yes	36	10	26	2.568	0.09
No	108	17	91		

*Note*: BUCY regimen (including cytarabine, busulfan, and cyclophosphamide) for myeloablative conditioning for 7 days. Cytarabine (8 g/msq) used on Day 1 to Day 2 and was infused for 3 h/day; busulfan (9.6 mg/kg) used from Day 3 to Day 5 and was infused for 8 h/day (divided into four infusion periods a day of 2 h each); and cyclophosphamide (3.6 g/msq) used from Day 6 to Day 7 and was infused for 3 h/day. Unit of house incoming in China is Yuan. Most clinical characteristics were similar between PWB Class 1 and Class 2 patients, except for the transplantation type.

### 3.4 Improved overall survival in PWB Class 2 patients

**FIGURE 2:**
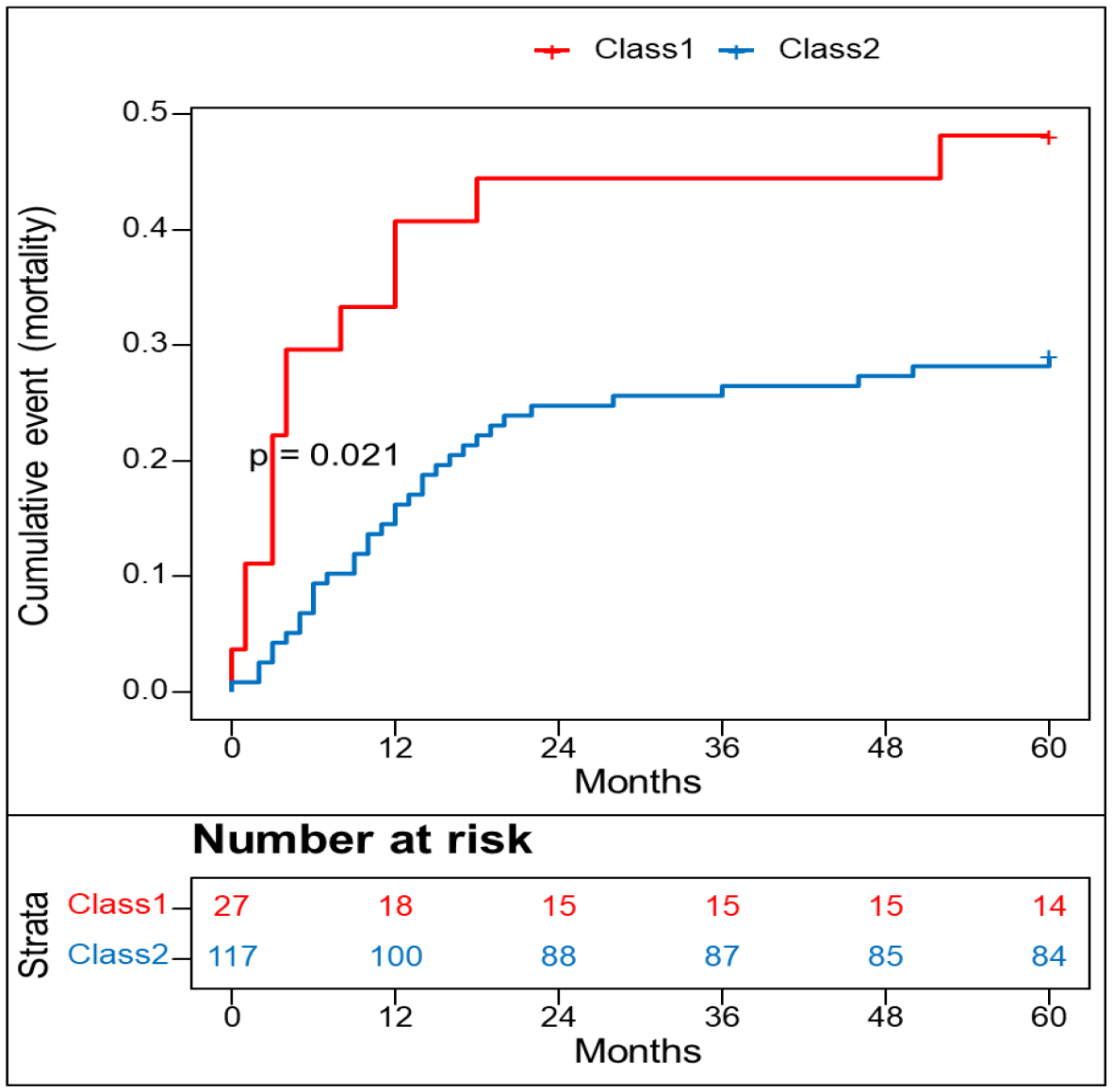
Two patient classes based on physical well‐being (PWB) score.

Kaplan–Meier curves demonstrated that patients in Class 2 had improved survival at the 60‐month follow‐up post‐HSCT. While Class 1 patients had a survival rate of 51%, 71% of Class 2 patients survived throughout the entire follow‐up period (Log‐rank *p* = 0.021). The median survival time was 52 months for Class 1, while it was not reached for Class 2 (Figure 2). In an unadjusted Cox model, Class 2 patients showed a 53% lower risk of mortality compared to Class 1 patients (HR [95% CI]: 0.47 [0.25–0.90], *p* = 0.023). After adjusting for demographic, clinical, and treatment factors, the risk of mortality remained lower in Class 2 than in Class 1 (Model 2, HR [95% CI]: 0.40 [0.17–0.96], *p* = 0.039). Moreover, after adjustment for significant factors from the univariate analysis, Class 2 showed similarly lower risk of death (Model 3, 0.36 [0.17–0.75], 0.005). These findings were consistent with the results from the survival analysis in the original dataset, which included missing values (Multivariate adjusted HR, Model 2 [95% CI]: 0.36 [0.17–0.77], *p* = 0.009, Model 3, 0.57 [0.29–1.12], *p* = 0.027) (Table 2).

**TABLE 2:** Multivariate analysis of LCA class and overall survival in HSCT patients.

	Imputated data		*p* value	Original data		*p* value
	PWB class			PWB class		
	Class 1	Class 2		Class 1	Class 2	
No. of cases (%)	13 (48.1)	34 (29.1)		9 (56.2)	38 (29.7)	
Unadjusted HR	1.00	0.47 (0.25–0.90)	0.023	1.00	0.38 (0.18–0.78)	0.008
Adjusted HR						
Model 1^a^	1.00	0.48 (0.25–0.92)	0.027	1.00	0.34 (0.16–0.72)	0.005
Model 2^b^	1.00	0.40 (0.17–0.96)	0.039	1.00	0.36 (0.17–0.77)	0.009
Model 3^c^	1.00	0.36 (0.17–0.75)	0.005	1.00	0.57 (0.29–1.12)	0.027

Abbreviation: HR, hazard ratio.

^a^ Model 1, adjusted for sex, age.

^b^ Model 2, adjusted for sex, age, marriage, house incoming, source of hospitalization, Previous history, diagnose, type of HSCT, disease status before HSCT, complication before HSCT, complication in HSCT, induced program.

^c^ Model 3, adjusted for hematopoietic reconstitution, disease status before HSCT, complication before HSCT, complication before HSCT (eye infection), complication before HSCT (renal insufficiency), complication before HSCT (cardiac insufficiency), complication before HSCT (central nervous leukemia), complication before HSCT (abnormal liver function), induced program, grouped by HCT‐CR.

## 4 DISCUSSION

**FIGURE 3:**
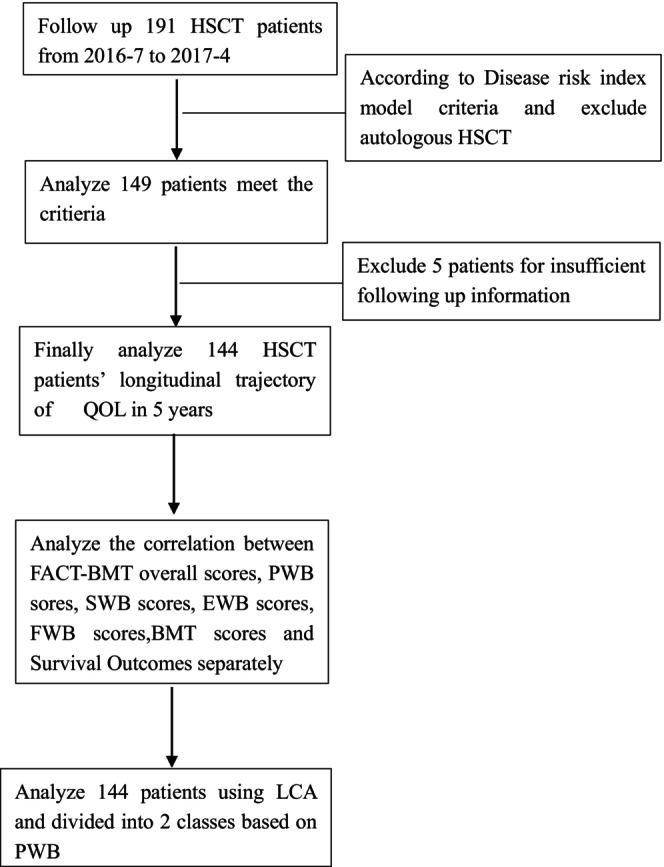
Study flow.

This retrospective study demonstrated the value of QOL parameters, especially the PWB in prediction of survival in long‐term following up HSCT patients (Figure 3).

HSCT remains the only curative option for various malignant hematological disorders. However, the high economic costs and inherent risks associated with the procedure have limited its widespread application. Therefore, developing a robust risk assessment protocol for individuals undergoing HSCT is of utmost importance. While existing scoring systems like the HCT‐CI and DRI have been proposed to assess HSCT risk, they primarily rely on objective parameters and may not fully capture patient‐reported data. In contrast, studies such as that conducted by William et al. have shown the crucial role of patient‐reported data in enhancing HSCT risk stratification.^9^

The evaluation of QOL in patients undergoing HSCT is crucial as it provides valuable insights into their physical and emotional well‐being.^21^, ^22^ In this study, we followed patient's QOL with FACT‐BMT for 5 years, and we discovered that the quality‐of‐life score reached a nadir after allogeneic HSCT and gradually improved within 6 months post‐HSCT. A higher QOL score over time was associated with improved survival at 5 years.

With the further analysis, we found that the trajectory of PWB over a five‐year follow‐up period closely mirrored the overall QOL trajectory. This observation aligns with previous research,^3^, ^23^ reaffirming the consistency of these findings in the field of HSCT. Our investigation into life quality evaluation revealed that HSCT patients commonly experienced physical discomfort, fatigue, nausea, pain, and sleepiness as their primary complaints. These symptoms are consistent with the well‐documented side effects associated with HSCT and its preparatory treatments.^24^

Notably, we observed an improvement in QOL relative to the patients' pre‐disease state by the 12‐month follow‐up, which is an encouraging finding. Despite the challenges and hardships faced during HSCT, many patients manage to regain a sense of normalcy and well‐being over time. Moreover, our study sheds light on the timing of emotional recovery in HSCT patients. While some studies have reported a delayed improvement in emotional status occurring after HSCT, our results show that declining physical health was associated with psychological recovery. This correlation underscores the complex interplay between physical and emotional well‐being in HSCT patients. Healthcare providers must recognize the importance of addressing both aspects of patients' health throughout the HSCT journey to optimize their overall QOL.

One of the most encouraging findings in our study is that most patients in our cohort were able to return to work and resume a normal lifestyle within 3–5 years after HSCT. This highlights the resilience and adaptability of HSCT patients, as well as the advancements in supportive care measures and post‐transplant rehabilitation programs. The ability to regain a sense of normalcy in life is a critical indicator of successful transplantation and improved QOL.

Furthermore, our study reveals a significant correlation between early QOL trajectories following HSCT and OS. Patients who demonstrated better life quality trajectories in the early post‐transplant period had improved OS rates. This observation emphasizes the importance of integrating QOL assessments into the routine care of HSCT patients as predictive indicators of long‐term outcomes. This finding aligns with our previous research^3^ and underscores the need to prioritize QOL as a critical outcome measure in HSCT.

In our study, we utilized a state‐of‐the‐art latent class model (LCA) to investigate the relationship between QOL and survival in HSCT patients. This advanced statistical approach allowed us to analyze high‐dimensional data without relying solely on predefined outcomes. The benefits of LCA have been demonstrated in various medical contexts, including studies on acute respiratory distress syndrome (ARDS), where it successfully classified patients into distinct sub‐phenotypes associated with survival outcomes. LCA‐based PWB trajectories were found to be correlated with survival outcomes. This correlation could be attributed to complications arising from chemotherapy or acute graft‐versus‐host disease, both of which can significantly impact a patient's physical condition.^24^, ^25^, ^26^ Additionally, exacerbations in physical conditions, coupled with emotional stress and limitations in self‐care, may contribute to a vicious cycle that compromises long‐term survival. This highlights the importance of early intervention and support for HSCT patients to break this cycle and improve their long‐term prognosis.^27^, ^28^

Furthermore, our findings emphasize the need for a comprehensive risk assessment protocol for HSCT patients that incorporates patient‐reported data. While existing scoring systems like HCT‐CI and DRI have their merits, the inclusion of PROs can provide a more holistic understanding of a patient's condition and improve risk stratification. Leveraging advanced statistical methods such as LCAs can further enhance our ability to predict outcomes and tailor interventions to the specific needs of HSCT patients.

Additionally, our findings suggest that early monitoring of QOL in the post‐HSCT period is crucial. Patients who exhibited more favorable QOL trajectories in the initial stages of their transplant journey had improved OS rates. This highlights the importance of early identification and intervention for patients who may be at risk of experiencing prolonged physical or emotional distress. Furthermore, integrating PROs into routine clinical assessments can improve patient‐centered care, enhance communication between patients and healthcare providers, and enable personalized treatment approaches.^29^

Despite the valuable insights provided by our study, there are several limitations that must be acknowledged. First, the QOL instrument used in our study was developed a century ago, and while it remains a valuable tool, it may lack some key elements relevant to modern society and the unique challenges faced by HSCT patients. This limitation highlights the need for continuous refinement and adaptation of QOL assessment tools to capture the evolving needs and experiences of patients. Second, the complexity of the numerous items in the QOL questionnaire may have made it less favorable for some patients to complete, leading to a high rate of missing data. Future research should focus on developing more concise and patient friendly QOL assessment tools to mitigate this issue.

## 5 CONCLUSION

Early‐stage PWB in HSCT patients may serve as a novel predictor of long‐term survival. This finding highlights the importance of considering QOL assessments and addressing PWB early in the HSCT journey. Further studies are warranted to explore the potential role of life quality as an interventional target in HSCT patients and to develop a more comprehensive approach to patient care in this complex medical journey.

## AUTHOR CONTRIBUTIONS

**Yin Lu:** Conceptualization (lead); data curation (equal); funding acquisition (lead); investigation (equal); methodology (equal); supervision (equal); writing – original draft (equal); writing – review and editing (equal). **Tao You:** Data curation (equal); formal analysis (lead); methodology (equal); writing – review and editing (equal). **Qin Ma:** Data curation (equal); formal analysis (equal); investigation (equal); project administration (equal). **Weijuan Wang:** Data curation (equal); investigation (equal); project administration (equal). **Jiaqian Qi:** Methodology (equal); supervision (equal); writing – review and editing (equal). **Pan Yang:** Formal analysis (equal); project administration (equal); writing – review and editing (equal). **Yaya Song:** Investigation (equal); project administration (equal). **Jia Chen:** Supervision (equal); writing – review and editing (equal). **Jianhong Fu:** Investigation (equal); supervision (equal). **Yongqin Ge:** Supervision (equal). **Xiaming Zhu:** Conceptualization (lead); funding acquisition (lead); project administration (equal); supervision (equal); writing – review and editing (equal). **Depei Wu:** Project administration (equal); supervision (equal).

## FUNDING INFORMATION

This study was supported by grants from Science and Technology Program of Suzhou. Construction and Research of early warning Model of Acute Graft Versus Host Disease from the Perspective of Precision Medicine (SYS2020112) and A Study on the Management of Intestinal Symptom clusters in Patients with Acute Graft Versus Host Disease under the Nursing Science Precision Health Model (SYKJ202104).

## CONFLICT OF INTEREST STATEMENT

The authors have no conflict of interest.

## ETHICS STATEMENT

This study had been approval by the Ethical committee of the First Affiliated Hospital of Soochow University (no.2017021).

## Supporting information


Table S1.

## Data Availability

Raw data is available if necessary.
