# Supplementary material for: Patient‐reported physical well‐being predicts good long‐term survival of hematopoietic stem cell transplantation
Source: Cancer Med. 2024 Jul 5;13(13):e7409. doi: 10.1002/cam4.7409 (PMC11225646; doi:10.1002/cam4.7409)
Supplement: Supplementary file 1 — Table S1. [file CAM4-13-e7409-s001.docx]

Supplemental table 1 Association of baseline clincal features with OS outcome

| variates |  | HR | 95%low | 95%upp | p |
| --- | --- | --- | --- | --- | --- |
| Age | Age | 0.99 | 0.97 | 1.02 | 0.4836 |
| Sex | Female=0 | 1 |  |  |  |
|  | male=1 | 0.93 | 0.52 | 1.66 | 0.8039 |
| Marriage | Spinsterhood=0 | 1 |  |  |  |
|  | married=1 | 0.81 | 0.45 | 1.45 | 0.481 |
|  | divorce=2 | 1.47 | 0.2 | 11.05 | 0.7061 |
| Residental address | City=0 | 1 |  |  |  |
|  | Town=1 | 0.91 | 0.4 | 2.04 | 0.8163 |
|  | county=2 | 0.77 | 0.32 | 1.86 | 0.5636 |
|  | village=3 | 0.75 | 0.38 | 1.48 | 0.4015 |
| Education | Master=0 | 1 |  |  |  |
|  | bachelor=1 | 0.73 | 0.09 | 5.61 | 0.761 |
|  | high school=2 | 0.55 | 0.07 | 4.09 | 0.5582 |
|  | Junior school=3 | 0.8 | 0.11 | 6.07 | 0.8298 |
|  | primary school=4 | 0.49 | 0.03 | 7.79 | 0.6108 |
| House incoming | More than 5001=1 | 1 |  |  |  |
|  | 4001-5000=2 | 0.61 | 0.2 | 1.88 | 0.3929 |
|  | 3001-4000=3 | 0.99 | 0.43 | 2.26 | 0.9831 |
|  | 2001-3000=4 | 0.94 | 0.41 | 2.14 | 0.8825 |
|  | Lower than 2000=5 | 0.84 | 0.38 | 1.84 | 0.6651 |
| Previous history | none=0 | 1 |  |  |  |
|  | Hypertension=1 | 0 | 0 | Inf | 0.9966 |
|  | Diabetes=2 | 1.21 | 0.17 | 8.81 | 0.8499 |
|  | Hepatitis B=3 | 1.52 | 0.47 | 4.91 | 0.4834 |
|  | others=4 | 0.9 | 0.32 | 2.51 | 0.8394 |
| Diagnosis | AML=1 | 1 |  |  |  |
|  | ALL=2 | 1.38 | 0.73 | 2.62 | 0.3207 |
|  | MDS=3 | 0.96 | 0.41 | 2.27 | 0.9348 |
|  | Lymphoma=4 | 3.11 | 0.73 | 13.28 | 0.1254 |
|  | CML=5 | 1.88 | 0.44 | 8 | 0.3957 |
| Disease risk index | Low-risk=1 | 1 |  |  |  |
|  | Middle-risk=2 | 2.68 | 0.36 | 20.08 | 0.3372 |
|  | High-risk=3 | 4.95 | 0.67 | 36.31 | 0.1158 |
| Disease status before HSCT | CR=0 | 1 |  |  |  |
|  | PR=1 | 0.9 | 0.22 | 3.75 | 0.888 |
|  | NR=2 | 2.31 | 0.71 | 7.5 | 0.1651 |
|  | Relapse=3 | 5.24 | 2.31 | 11.89 | <0.0001 |
|  | CRi=4 | 1.91 | 0.16 | 8.7 | 0.8618 |
|  | Others=5 | 0 | 0 | Inf | 0.9971 |
| Grouped by HCT-CR | Low-risk=1 | 1 |  |  |  |
|  | middle-risk=2 | 0 | 0 | Inf | 0.9974 |
|  | high-risk=3 | 2.02 | 1.13 | 3.59 | 0.0168 |
|  | Very highe-risk=4 | 0 | 0 | Inf | 0.9974 |
| Induced programme | BUCY=1 | 1 |  |  |  |
|  | Decitabine+BUCY =2 | 1.15 | 0.53 | 2.47 | 0.7231 |
|  | Others=4 | 3.11 | 1.1 | 8.75 | 0.0317 |
| Type of HSCT | Matched-related=1 | 1 |  |  |  |
|  | Haplo-=2 | 1.33 | 0.71 | 2.49 | 0.3769 |
|  | Unrelated-=3 | 0.64 | 0.18 | 2.23 | 0.4832 |
| Hematopoietic reconstitution | Yes=1 | 1 |  |  |  |
|  | No=2 | 0.65 | 0.26 | 1.65 | 0.3634 |
| Complication before HSCT | No=0 | 1 |  |  |  |
|  | Yes=1 | 2 | 1.11 | 3.61 | 0.0209 |
| Complication before HSCT(Eye infection) | No=0 | 1 |  |  |  |
|  | Yes=1 | 148 | 9.26 | 2366.27 | 0.0004 |
| Complication before HSCT(Renal insufficiency) | No=0 | 1 |  |  |  |
|  | Yes=1 | 49 | 5.1 | 471.07 | 0.0008 |
| Complication before HSCT(Abnormal liver function) | No=0 | 1 |  |  |  |
|  | Yes=1 | 7.28 | 1.71 | 30.94 | 0.0071 |
| Complication before HSCT ( Central nervous leukemia) | No=0 | 1 |  |  |  |
|  | Yes=1 | 3.49 | 1.25 | 9.74 | 0.0169 |
| Complication before HSCT (Cardiac insufficiency) | No=0 | 1 |  |  |  |
|  | Yes=1 | 5.44 | 1.3 | 22.76 | 0.0203 |
| Complication before HSCT (Pulmonary infection) | No=0 | 1 |  |  |  |
|  | Yes=1 | 1.36 | 0.66 | 2.8 | 0.4086 |
| Complication before HSCT (Other) | No=0 | 1 |  |  |  |
|  | Yes=1 | 1.05 | 0.15 | 7.65 | 0.9579 |
| Complication in HSCT | No=0 | 1 |  |  |  |
|  | Yes=1 | 2.88 | 0.4 | 20.86 | 0.2954 |
| GVHD | No=0 | 1 |  |  |  |
|  | Yes=1 | 2.07 | 0.82 | 5.23 | 0.1234 |

Note：CR: Complete remission; PR: Part remission; NR: Non-remission; CRi: Hypoplasia; BUCY regimen (including cytarabine, busulfan and cyclophosphamide) for myeloablative conditioning for seven days. Cytarabine (8 g/msq) used on day 1 to day 2 and was infused for 3 h/day; busulfan (9.6 mg/kg) used from day 3 to day 5 and was infused for 8 h/day (divided into four infusion periods a day of 2 h each); and cyclophosphamide (3.6 g/msq) used from day 6 to day 7 and was infused for 3 h/day.
